# Direct imaging of plasma waves using ultrafast electron microscopy

**DOI:** 10.1063/4.0000044

**Published:** 2020-12-24

**Authors:** Shuaishuai Sun, Xiaoyi Sun, Daniel Bartles, Elliot Wozniak, Joseph Williams, Peng Zhang, Chong-Yu Ruan

**Affiliations:** 1 Department of Physics and Astronomy, Michigan State University, East Lansing, Michigan 48824, USA; 2 Department of Electrical and Computer Engineering, Michigan State University, East Lansing, Michigan 48824, USA

## Abstract

A femtosecond plasma imaging modality based on a new development of ultrafast electron microscope is introduced. We investigated the laser-induced formation of high-temperature electron microplasmas and their subsequent non-equilibrium evolution. Based on a straightforward field imaging principle, we directly retrieve detailed information about the plasma dynamics, including plasma wave structures, particle densities, and temperatures. We discover that directly subjected to a strong magnetic field, the photo-generated microplasmas manifest in novel transient cyclotron echoes and form new wave states across a broad range of field strengths and different laser fluences. Intriguingly, the transient cyclotron waves morph into a higher frequency upper-hybrid wave mode with the dephasing of local cyclotron dynamics. The quantitative real-space characterizations of the non-equilibrium plasma systems demonstrate the feasibilities of a new microscope system in studying the plasma dynamics or transient electric fields with high spatiotemporal resolutions.

## I. INTRODUCTION

Incorporating femtosecond electron pulses into electron microscopes has enabled ultrafast electron microscopy (UEM) of solids and macromolecules with unprecedented temporal resolutions.^1–4^ With their ingrained field sensitivity to the local electrodynamics, ultrafast electron imaging of plasma matters or the metamaterials represents yet another frontier.^5–11^ In particular, retrofitting high-brightness electron sources into a UEM system may boost the sensitivity for resolving dynamics on the fs–nm scale. While probing transient plasma fields with an electron beam is a well-known technique,^12–16^ the direct field imaging approach with high spatial resolution is envisioned to have direct implications in microplasma diagnostics and probing ultrafast charge transport dynamics across material interfaces, such as electron motion in microcavity plasma and nanoscale vacuum channel electronics,^5–7^ local field enhancement,^8^ plasmon-induced hot carrier generation,^9^ strong-field photoemission,^10^ and quantum tunneling.^17^

As an ultimate many-body system with long-range interactions, plasma hosts a variety of instabilities and plasma wave modes.^18,19^ Mitigating the instability growth and controlling plasma waves have been central in designing advanced accelerators with high-density beams^20,21^ and systems for magnetic or inertial confinement fusion.^22,23^ On the other hand, there have also been important recent progresses in identifying new forms of plasma instabilities and wave excitations in extreme settings (temperature, density, or magnetic field) of both the laboratory^18,23^ and space plasmas.^24,25^ Understanding the transient nonlinear plasma dynamics and how plasma waves evolve from initial conditions is of fundamental importance to both the basic physics and advanced technological developments. In a dilute setting, the cyclotron waves occur under a strong magnetic field as effective conduits for transporting particles and energy,^19^ whereas hybrid modes emerging in high-density systems and at high temperatures^22,26^ become acute in strongly confined miniaturized plasma sources. Despite extensive studies, the excitation process of the upper hybrid waves and the temporal evolution of the wave dynamics are not adequately understood. While relying heavily on sophisticated numerical simulations,^27^ experimental capabilities of directly observing and probing plasmas are critical in studying the non-equilibrium processes of plasma evolution and its associated initial conditions, such as instability seedings and the dynamical structures.

Here, we report the study of laser-induced electron plasmas from copper grid surfaces under magnetic field and their subsequent non-equilibrium evolution by a radio frequency (RF) compressed UEM with high-brightness electron sources. We developed a straightforward imaging approach to extract the dynamical electric field profile of plasma and quantitatively determine the density modulations, wave modes and frequencies, particle number, and effective temperature. The key finding is that the bunched energetic plasma emissions under a magnetic field manifest in cyclotron echoes and induce plasma wave oscillations underpinned by microscopically reversible cyclotron dynamics in warm plasma systems. The relatively long decay time of echoes features the weakly collisional nature and the ability of the laser-generated plasma to support new wave modes out of equilibrium. We show that the spontaneously generated plasma waves finally turn into the upper-hybrid modes expected of a magnetized plasma under an equilibrium condition and are magnetically tunable over a cyclotron dephasing time. This work represents a prototype for demonstrating the robustness of an ultrafast field-imaging protocol for extracting the dynamical field profile from micro-structured plasma objects.

## II. EXPERIMENTAL APPROACH

### A. UEM setup

**FIG. 1.**
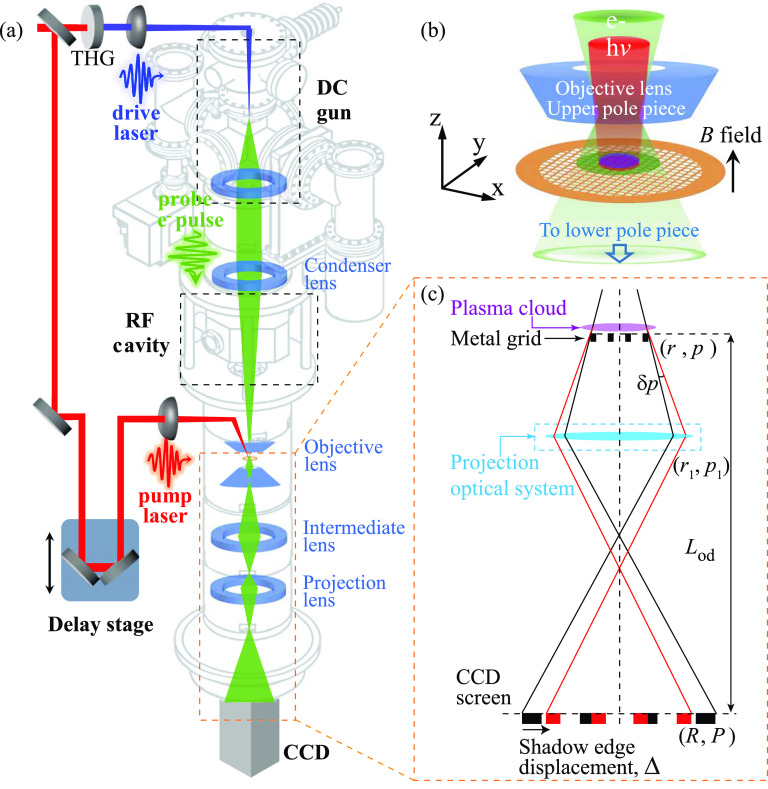
Ultrafast electron microscope system for imaging plasma dynamics. (a) Schematic of the UEM system. (b) Scale-up view of the specimen chamber showing the incident pump laser (red) for generating the plasmas (purple) and the probe electron beam (green). (c) Ray diagram depicting the transmitted probe beam to cast a shadow of the copper grid onto the CCD screen, with black and red rays representing the trajectories without and with the plasma field present. For details, see the text.

The experiments were performed in a new UEM system. To implement ultrafast imaging, key modifications are made in a Hitachi H800 transmission electron microscope (TEM) to outfit it into a UEM, as outlined in Fig. 1(a). In a significant departure from the earlier approach,^1^ the new UEM reported here employs a high-flux photo-electron gun, operated near the virtual cathode limit, to boost the beam brightness.^28^ To gain sensitivity to the field, low-energy beams (25 keV) and ≈10^5^ electrons/pulse at 10 kHz are employed. The spatiotemporal resolutions are optimized utilizing an RF cavity functioning as a longitudinal focusing lens.^29,30^ With the set initial pulse parameters at the cathode, a pulse duration of ≈100 fs is accomplished at the object plane with pulse compression by RF cavity; alternatively, via minimizing the energy spread (1.3 eV), ≤10 nm spatial resolution can be achieved; see the supplementary material Fig. 3. For imaging the dynamics, we use an optical stage to define the delay (*t*) sequences following the pump–probe protocols.^1^

Here, the micro-structured plasma sources are conveniently generated from the top surfaces of the copper grid held within the objective pole pieces upon pump laser (∼50 fs, 800 nm) incidence along the optical axis (*z*); see Fig. 1(b). The relatively large footprint of the Gaussian laser profile (sigma-width $\sigma_{r}\;\approx$ 110 *μ*m) initially creates a perforated thin disk of plasma cloud. To study the magnetically confined plasmas, we set up the magnetic field (B = 0–1 T) by applying a current (*I_obj_*) to the objective lens of the TEM.

### B. Imaging field profile

The modulation of the probe beam coming off the specimen by the plasmas (corresponding to a transverse momentum transfer δp) creates the necessary contrast to image the plasma fields. To maintain the sensitivity to probe δp for imaging plasmas, we operate the microscope in the out-of-focus mode.^31^ A simplified ray diagram illustrating the processes is presented in Fig. 1(c), where we use ***r*** and ***R*** to represent the coordinates of the plasma and the image formed at the CCD. More details about the projection system, which controls the magnification *M* and the images with several lenses, are elaborated in the supplementary material. The copper grid is optically thick; hence, the out-of-focus mode setting effectively casts a shadow of the grid onto the CCD. The field effects, or δp introduced locally, are examined via comparing the patterns formed under the field perturbations with the one (reference frame, *t *<* *0) formed without the fields present. In Fig. 1(c), we use rays in black and red to describe the respective unperturbed and perturbed image formation, where the difference between the two describes the displacement vector $\Delta \left(R\right)$. Experimentally, the displacement vector is determined by fitting edges of the grid bar images before and after applying laser pulses using an error function. We can easily show (for details, see the supplementary material) that the $\Delta \left(R\right)$ is directly proportional to $\delta p\left(r\right)$ under a mapping R=Mr regardless of the optical settings,

$$\Delta =\frac{\delta p}{P_{0}}L_{\mathrm{cam}},$$ (1)

where $P_{0}$ represents the beam momentum and $L_{\mathrm{cam}}$ represents the specimen-camera distance. Given the small profile size along *z* (Δz), the relevant dynamics is averaged out over the short transit time (≲1 ps) of the probe, and the displacement vector $\Delta \left(R\right)$ is mainly sensitive to the electric field in the transverse directions,

$${\;E}_{r}\left(r;t\right)=C_{1}\Delta \left(R;t\right)$$ (2)

with $C_{1}=\frac{P_{0}V_{e}}{{eL}_{\mathrm{cam}}\Delta z}$, where $V_{e}$ represents beam velocity; see the supplementary material for details. The important advantage of our approach is the direct field imaging assisted by mesh-indexing based on Eq. (2), which does not depend on the details of the TEM imaging optics that involve the objective (to provide *B*) and several projection lenses.

### C. Characterizing plasma size

We anticipate that the plasma clouds generated here adopt a broad energy distribution. This is informed by multiple empirical data of photoemission obtained from grid surfaces.^32,33^ While several effects (the initial states, nonequilibrium electrons, and the space charges) are attributed to the energy broadening from multiphoton ionization and optical field tunneling,^10,17,34,35^ for the purpose of modeling here, we treat the earlier results with the thermal Maxwell–Boltzmann (MB) distribution to deduce the effective $T_{e}$. We show that the MB model captures the experimental spectra very well and deduce $T_{e}$ of 5500 and 12 200 K under the laser intensity *P* of 1 × 10^9^ and 1.2 × 10^11^ W/cm^2^, respectively; see Ref. 32 and the data reproduced in the supplementary material. Since here we target *P* ≈ 10^12^ W/cm^2^, we expect even higher $T_{e}$ to be developed.

We assume that the MB plasma cloud generated here has a Gaussian profile. To construct an effective model for the thin plasma disk emitted, we describe the density distribution in the cylindrical coordinates,

$$\rho \left(r,z;t\right)={en}_{e}\left(r,z;t\right)=eN_{e}h\left(z;t\right)g\left(r;t\right),$$ (3)

where $N_{e}$ is the total number of electrons in the plasma system. The distribution functions along ***z*** and ***r*** directions are $h\left(z;t\right)=\frac{1}{\sqrt{2\pi }\sigma_{z}}e^{-\frac{z^{2}}{2\sigma_{z}^{2}}}$, $g\left(r;t\right)=\frac{1}{2\pi \sigma_{r}^{2}}e^{-\frac{r^{2}}{2\sigma_{r}^{2}}}$, where $\sigma_{z}$ and $\sigma_{r}$ are the corresponding width in each direction. At a given delay *t*, the displacement profile is mapped via $\delta p_{r}$ under a plasma field $E_{r}\left(z,r;t\right)$. For a Gaussian distribution, we obtain

$$E_{r}\left(z,r;t\right)=\frac{eN_{e}h\left(z;t\right)}{2\pi \varepsilon_{0}r}\left(1-e^{-\frac{r^{2}}{2\sigma_{r}^{2}}}\right)$$ (4)

with $\varepsilon_{0}$ representing the vacuum permittivity. Then, the momentum transfer is calculated through ${\delta p}_{r}=\int_{z_{-}}^{z_{+}}eE_{r}\left(z,r;t\right)dt.$ As the plasma is largely bound within $\pm 2\sigma_{z}$, i.e., $\int_{-2\sigma_{z}}^{2\sigma_{z}}h\left(z\right)dz\;\;∼\;1$, we integrate out the *z*-dependence over the short transit time of the probe (maximally a few ps; see the supplementary material Table I) and derive from Eqs. (2) and (4)

$$\Delta \left(R\right)=C_{2}N_{e}\left(1-e^{-\frac{r^{2}}{2\sigma_{r}^{2}}}\right)/r$$ (5)

with $N_{e}$ the total number of electrons in the system and $C_{2}=\frac{{e^{2}L}_{\mathrm{cam}}}{2\pi \varepsilon_{0}\gamma m_{e}V_{0}^{2}}$, where $\varepsilon_{0}$, γ, and $m_{e}$ represent the vacuum permittivity, relativistic Lorentz factor, and the electron rest mass, respectively.

The collective wave responses can be extracted from Eq. (5). Since $N_{e}$ is conserved, the plasma density modulations must be conveyed through the modulations in $\sigma_{r}$ (see the supplementary material), which is effectively a longitudinal (Langmuir) wave with

$$\sigma_{r}\left(t\right)=\sigma_{r0}\left(t\right)+\sigma_{p}\;\text{cos}\;\left(2\pi f_{p}t+\phi \right),$$ (6)

where $\sigma_{p},\;f_{p}$, and ϕ are the amplitude, frequency, and phase angle of the plasma oscillation, respectively. The $\sigma_{r0}\left(t\right)$ term describes the non-oscillatory plasma expansion, which, for practical purposes here, can be expressed as

$$\sigma_{r0}\left(t\right)=A+v_{0}t$$ (7)

with $v_{0}$ representing the expansion velocity.

## III. RESULTS AND ANALYSIS

The freedom to provide a broad range of magnification in our UEM system allows field imaging of plasma dynamics at different scales.

### A. Collective plasma wave dynamics

**FIG. 2.**
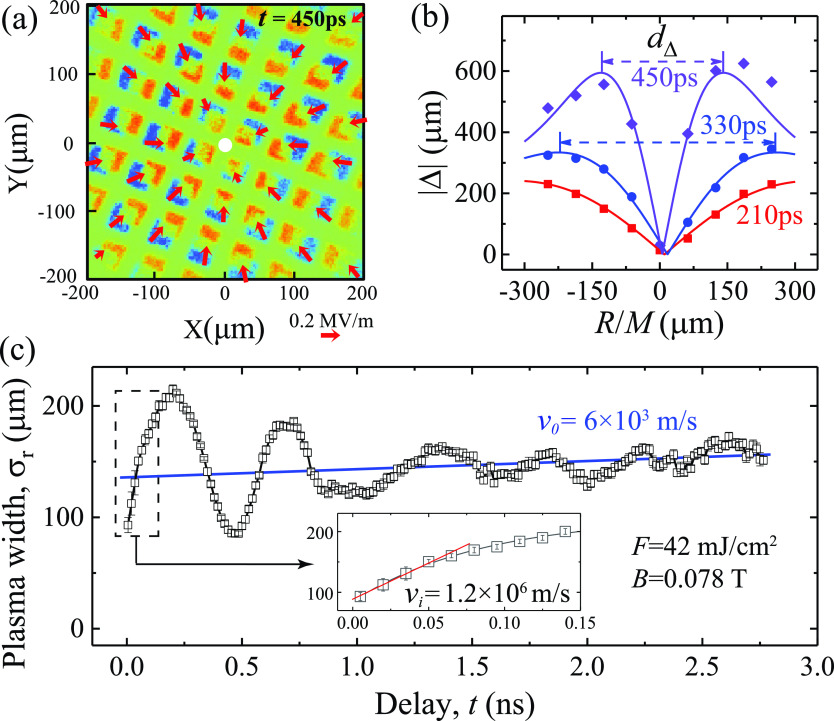
Plasma profile evolution. (a) Typical difference image of the grid (450 ps) showing the edge displacement Δ and reconstructed electric field (marked by red arrows) along radial directions. (b) Extracted displacement profiles at *t *=* *210, 330, and 450 ps. The ridge-to-ridge distance (*d*_Δ_ = 3.17 $\sigma_{r}$) is proportional to the transverse plasma size. The solid lines show the fits with plasma width $\sigma_{r}$ of 211 ± 2, 153 ± 2 and 86 ± 2 *μ*m, respectively. (c) Dynamics indicating plasma oscillations, where the blue baseline shows a slow plasma cloud expansion under magnetic confinement. The inset shows the initial space-charge-driven expansion at a much higher velocity. All data here were obtained at *F *=* *42 mJ/cm^2^ and *B *=* *0.0776 T.

We first examine the collective responses that span the entire plasmas using a relatively low magnification (*M* ∼ 30–50). The experiments are set at *F *=* *42 mJ/cm^2^ and *B *=* *0.078 T. The difference image deduced from comparing plasma-on (*t *>* *0) and plasma-off (*t *<* *0) images, i.e., $\Delta I\left(R;t\right)=I\left(R;t\right)-I\left(R;t<0\right)$ presented in Fig. 2(a) with *t *=* *450 ps, shows a clear radial pattern of vector displacement $\Delta \left(R;t\right)$. The vector electric fields conveniently tracked at mesh interconnects computed by applying Eq. (2) are marked by the red arrows. The maximum transverse electric field here is ∼0.2 MV/m. Such displacement and field patterns, conveniently tracked at grid's shadow edges, exhibit the features of radial breathing oscillation along the direction of ***R***, which one can follow at different delays; see the supplementary material Fig. 9. Figure 2(b) shows the results of $\left|\Delta \left(R;t\right)\right|$ at 210, 330, and 450 ps mapping to the object plane. The cusp-like profiles in $\left|\Delta \right|$ plotted here provide a measure of plasma transverse size directly based on the ridge-to-ridge distance ($d_{\Delta }$= 3.17 $\sigma_{r}$). Here, the data can be well fitted with analytical profiles (solid lines) based on Eq. (5) to get the plasma width $\sigma_{r}$ = 211 ± 2, 153 ± 2, and 86 ± 2 *μ*m, respectively. This demonstrates that indeed the MB Gaussian model describes the plasma profiles well. We then extend this approach to the entire observation time window. The oscillations $\Delta \left(R;t\right)$ are now directly mapped into the source plane (***r***) in terms of $\sigma_{r}\left(t\right)$, as depicted in Fig. 2(c), which are well described by Eq. (6) based on longitudinal (Langmuir) oscillations. We also capture the expansion of the plasma cloud according to Eq. (7). The expansion starts at a higher velocity $v_{i}=$ 1.2 × 10^6^ m/s reflecting a strongly space-charge-driven dynamics initially; see the inset. The velocity then rapidly decreases in just 100 ps and turns into a terminal speed of $v_{0}=$ 6 × 10^3^ m/s. The sharp turnaround showcases the magnetic confinement in the system that effectively redirects the space-charge-driven dynamics into plasma oscillations. We note that the results obtained here are deduced by keeping $N_{e}$ = 2.25 × 10^6^ as constant in the fitting, with the exception of the first 20 ps where the $N_{e}$ increases with time. This indicates that after 20 ps, the plasma cloud is entirely detached from the grid surfaces.

**FIG. 3.**
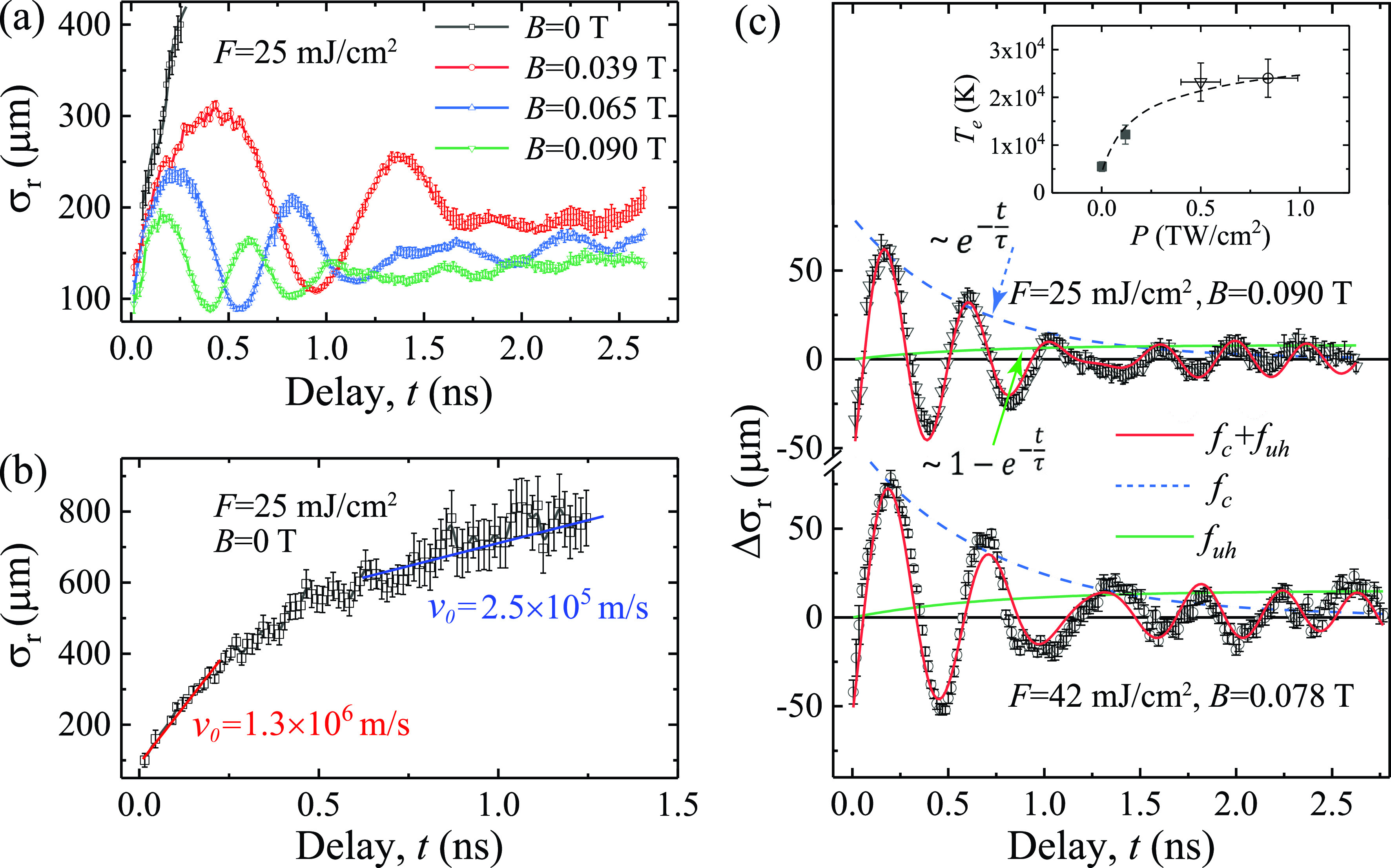
Transformations of plasma Langmuir oscillations into upper-hybrid resonance waves. (a) Plasma oscillations at *F *=* *25 mJ/cm^2^ under different magnetic fields. (b) Expansion of plasma cloud at *F *=* *25 mJ/cm^2^ without magnetic field. (c) The transition of plasma oscillations into upper-hybrid waves at two laser fluences. The inset summarizes the $T_{e}$ deduced from different laser intensities (*P*) based on Ref. 32 (solid symbols) and the studies here (open symbols). The experimental data are fitted (in red solid line) with $\Delta \sigma \left(t\right)=A_{c}\;\text{sin}\;\left(2\pi f_{c}+\phi_{c}\right)e^{-\frac{t}{\tau }}+A_{uh}\;\text{sin}\;\left(2\pi f_{uh}+\phi_{uh}\right)\left(1-e^{-\frac{t}{\tau }}\right)$, where $A_{i}$ and $\phi_{i}$ represent the amplitude and phase of the two plasma waves, respectively. The dashed blue line and solid green line outline the decay and the rise of the waves at $f_{c}$ and $f_{uh}$ with the same time constant τ, respectively.

The longitudinal breathing mode identified here is directly coupled to a density oscillation (see the supplementary material), which are expected to associate with the density ($n_{e}$)-dependent plasma frequency $f_{p}=\frac{e}{2\pi }\sqrt{\frac{n_{e\;}}{m_{e}\varepsilon_{0}}}$. Curiously, here the wave mode (50–700 ps) tracks very well with the cyclotron frequency $f_{c}=\frac{eB}{2\pi m_{e}}$ independent of $n_{e}$. To understand how magnetized plasma cloud transforms into plasma waves, we systematically investigate the plasma wave responses at different magnetic fields while keeping *F* as constant (25 mJ/cm^2^), as reported in Fig. 3(a). It clearly shows that the gradual reduction of both the oscillation amplitude and the mean plasma size is solely linked to an increasing *B*, while the impulsive initiation of the plasma cloud shown in the first 100 ps evolution by Coulomb repulsion remains unchanged—this is a remarkable demonstration of our field-imaging protocol amidst the significant change in *B* that alters the imaging optics entirely. We confirm that, in all confined cases, the initial density oscillation frequency is consistent with $f_{c}$, indicating that the initial plasma wave is directly caused by the magnetic confinement underpinned by the particle cyclotron motions. To see how the system evolves without the confinement, we single out the *B *=* *0 case in Fig. 3(b). The cloud continues to expand well beyond its initial size and eventually reaches a steady state where the expansion is no longer driven by Coulomb explosion but by the internal velocity dispersion of the cloud. The unconfined expansion here highlights the impulsive initiation by the space charges that when held under a strong magnetic field transforms into collective responses in wave instabilities.

While in all confined cases the initial oscillation frequency is consistent with $f_{c}$, the oscillations shift to a new higher frequency mode at the late stage. This is more closely examined through the fittings outlined in Fig. 3(c), where we determine the new wave modes to be at 2.75 and 2.58 GHz, increased from the initial $f_{c}$ at 2.53 and 2.17 GHz at two fluences (25 and 42 mJ/cm^2^). A rational explanation is that the higher frequency mode is in fact the upper-hybrid modes at $f_{uh}=\sqrt{f_{p}^{2}+f_{c}^{2}}$, anticipated from a warm magnetized system under the steady-state condition.^19^ Following this, we fit the data obtained at two different fluences with a model (thick red solid line) that assumes that the decay of the initial cyclotron wave at $f_{c}$ (thin blue dashed line) is directly coupled with the rise of the new hybrid wave mode at $f_{uh}$ (thin green solid line) at a time constant τ (1/*e*). The experimental data are fitted (in red sold line) with

$$\Delta \sigma \left(t\right)=A_{c}\;\text{sin}\;\left(2\pi f_{c}+\phi_{c}\right)e^{-\frac{t}{\tau }}+A_{uh}\;\text{sin}\;\left(2\pi f_{uh}+\phi_{uh}\right)\left(1-e^{-\frac{t}{\tau }}\right),$$ (8)

where $A_{i}$ and $\phi_{i}$ represent the amplitude and phase of the two plasma waves, respectively. τ is determined to be 600±100 and 700±150 ps, respectively. Hence, our measurements witness a delicate non-equilibrium process that transforms an initial system dominated by the particle-like dynamics to a more stable magnetized plasma wave at the hybrid frequency, in which the intriguing initial plasma wave state is a transition state.

To verify that the eventual state is indeed the upper-hybrid mode, we calculate $f_{p}$ from $f_{uh}$ under this assumption and deduce the electron density $n_{e\;}[=m_{e}\varepsilon_{0}{\left(2\pi f_{p}/e\right)}^{2}]$—a technique widely used to survey $n_{e}$ of space plasmas.^25,26^ We determine $f_{p}=1.1\pm$ 0.1 GHz for the waves at *F *=* *25 mJ/cm^2^, hence a $n_{e}$ of 1.5±0.3 × 10^16^ m^−3^. To independently find out $n_{e}$, we resort to the time-of-flight data in Fig. 3(b), where the terminal velocity $v_{0}$ = 2.5±0.5 × 10^5^ m/s reflects a thermal expansion, which we apply to deduce the unconfined $\sigma_{z}$ at the relevant late stage (see the supplementary material). Based on the well-defined protocol [see Eq. (5)], we deduce the $N_{e}$ and $\sigma_{r}$ directly from the experimental profiles, and with the $\sigma_{z}$ obtained here, we determine the $n_{e}$ = 1.7±0.4 × 10^16^ m^−3^. The two $n_{e}$ values do agree reasonably well, thus affirming that the eventual state is the upper-hybrid wave expected of the plasma density.

For describing the behavior from a broad energy distribution, we estimate the effective temperature of the plasma $T_{e}$ to be ≈25 000 K, by treating the system with a Maxwell–Boltzmann (MB) distribution where the flux velocity $v_{F}=0.282{\left(\frac{2k_{B}T_{e}}{m_{e}}\right)}^{1/2}$ (see the supplementary material). The $T_{e}$ determined here is consistent with other studies that also show a broad energy distribution in the emitted electrons from grid surfaces upon intense laser irradiation.^32,33^ The results are fitted empirically with the MB distribution to estimate $T_{e}$ in these systems; see Ref. 33 and the data fitting^32^ reproduced in the supplementary material and plotted in the inset of Fig. 3(c).

### B. Initial plasma seeding dynamics and cyclotron echo

**FIG. 4.**
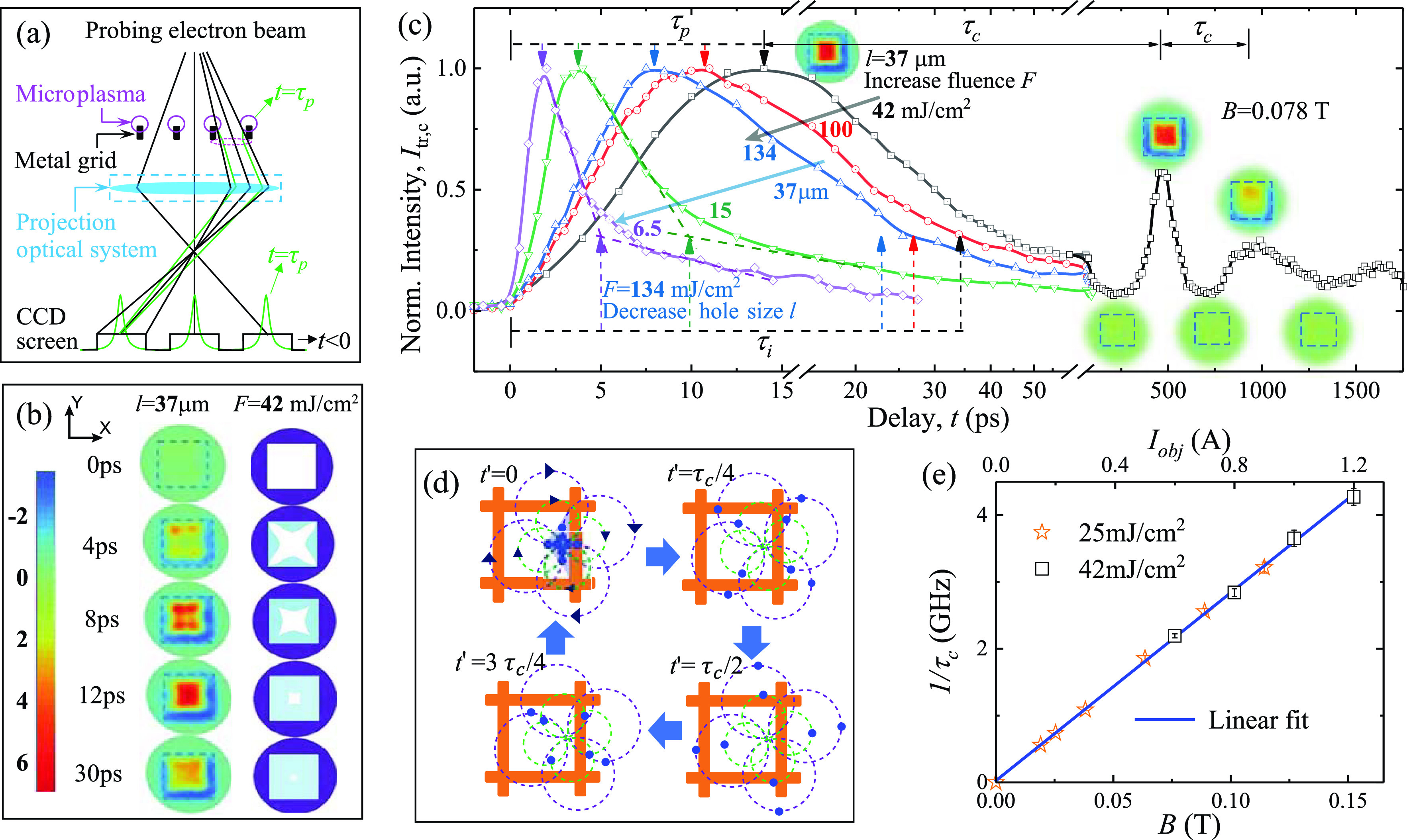
Local plasma dynamics and cyclotron bunching echoes. (a) Schematic diagram showing the plasma lensing engineered by the local inhomogeneous fields from the photoemitted microplasmas at the early stage. (b) Time-resolved images (subtracted by the negative frame, left panel) and reconstructed local plasma evolution (sky blue, right panel) in the early stage. The dashed square represents the edge of the mesh cell with a width (*l*) of 37 *μ*m. (c) The evolution of normalized intensity $I_{tr,c}$ at the center region. The peaking time ${\;\tau }_{p}$ and inflection time $\tau_{i}$ characterize the initial evolution (0–50 ps) for results obtained with different pump fluences (*F*) and hole sizes (*l*). The echoes of the initial intensity maximum are obtained at the period $\tau_{c}$. (d) Cartoon depiction of cyclotron rebunching. The dashed lines show the individual particle cyclotron trajectories with different initial velocities (arrows and colors represent direction and amplitude). The locations of the particles are tracked with blue dots. Over the period $\tau_{c}$, the electrons return to the initial locations of the bunched emission. Here, *t′* = *t′*${-\tau }_{p}$. (e) The frequency deduced from (1/$\tau_{c}$) as a function of *B* provided through the objective lens current (*I_obj_*).

After tackling the collective wave responses that span the entire plasmas at a later time, it is essential to understand the initial local seeding process of plasmas and how it evolves into the transient cyclotron wave state. Here, we decrease the field of view by a factor of 10 (*M *=* *500) and focus on a single mesh cell. At the early stage, the pulsed microplasma emission is initiated by the pulsed irradiation and assisted by the near-surface space-charge repulsion for launching into free space. We anticipate the pulse to be stretched by the velocity dispersion developed during the emission as shown previously.^16,36^ Here, the random directions and broad energy distribution of the emitted electrons allow the pulsed microplasmas expand into the free space above the mesh cell and be captured by the electron imaging technique, as depicted in Fig. 4(a). The results, as presented in the left panels of Fig. 4(b), show such dynamics using the difference images, $\Delta I\left(X,Y;t\right)=I\left(X,Y;t\right)-I\left(X,Y;t<0\right)$, focusing at the early delays (*t *=* *0–30 ps). Hot spots (as those colored in red) are observed initially at the cell corners, reflective of the inhomogeneous initial microplasma emissions preferentially from the side surfaces of the grid rather than at the interconnects. Based on the understanding of the optics posed by the plasma fields [see Fig. 4(a) and the supplementary material for more details], the transient profiles of the microplasmas are reconstructed in the right panels (sky blue). As the microplasmas extend into the hole region, the hot spots move toward the center. The increased repulsion brings the crossover point of the transmitted rays to the image plane [Fig. 4(a)], resulting in a strong plasma lensing effect at ${\;\tau }_{p}$ ≈ 12 ps.

We can replicate such dynamical lensing profiles using different pump fluences (*F*) or different sizes of mesh hole (*l*). A simple way to track the evolution is by examining the intensity at the center of the mesh hole, $I_{tr,c}\left(t\right)$, which are plotted in Fig. 4(c). The general evolution is characterized by a rapid rise of $I_{tr,c}$ followed by a ∼3 times slower decay. The rise and decay of $I_{tr,c}\left(t\right)$ here reflect the focusing and defocusing of the probing beam that take place as the microplasma fronts gradually close in the cell. At $\tau_{p}$, the local fields focus the beam at the CCD; whereas at the inflection point $\tau_{i}$, the focusing effect has decreased and so $I_{tr,c}$ is mainly driven by the collective plasma dynamics in a largely smooth field profiles, as indicated in Fig. 4(b). For the specific setting (*l *=* *37 *μ*m, *F *=* *42 mJ/cm^2^), we identify $\tau_{p}$≈ 13 ps and $\tau_{i}$≈ 35 ps. By raising *F* to 134 mJ/cm^2^, we obtain shorter timescales in $\tau_{p}$ (≈8.5 ps) and $\tau_{i}\;($≈23.5 ps), indicating the increased expansion velocities due to higher density of space charges that more effectively propel the microplasma bunches into the free space. More interestingly, it is further demonstrated that $\tau_{p}$ and $\tau_{i}$ both scale linearly with the cell size (*l*) and the different $I_{tr,c}\left(t\right)$ obtained under the same *F* can collapse into a single curve. The latter implies that the dynamics within the cell are self-similar on the different *l* scales; see the supplementary material for complete data analyses.

Applying a constant magnetic field (*B*), we observe intriguing revivals of the plasma lensing effect at an interval $\tau_{c}$, as shown on the right side of Fig. 4(c). As the lensing effect is specifically tailored by the inhomogeneous profiles at microplasma seeding, this means the plasma dynamics under the magnetic field retains the memory of the initial conditions. This interesting phenomenon can be understood by that, despite the different trajectories the particles undertake in the ensuing cyclotron motions, the particle systems must return to their initial positions after the cyclotron period $\tau_{c}=\frac{1}{f_{c}}$, where under a homogeneous *B* field the cyclotron frequency $f_{c}$ is the same for all particles. Locally due to Coulomb repulsion, the microplasma bunches are launched into the free space with a boosted center-of-mass (CoM) velocity from all sides of the cell and establish the strong signal $I_{tr,c}$ at first. Then, the micro-bunches at their individual CoM frames will undergo circular cyclotron motion perpendicular to the static *B* field. However, the velocity dispersion from the thermal broadening effect spreads the trajectories of particles within the micro-bunches. This causes the $I_{tr,c}$ signal to decay rapidly and establishes a more homogeneous cloud. At F = 42 mJ/cm^2^, the CoM speed of the micro-bunches (v_CoM_ ≈ 0.5 × 10^6^ m/s) gives a mean cyclotron orbit radius $r_{c}\;\approx \;40$*μ*m under B = 0.078 T (see the supplementary material), much smaller than the plasma cloud transverse size (FWHM) of ≈340 *μ*m. We point out that the magnetic field associated with the returning currents is negligible (<10^−5^ T) compared to the applied field. Hence, we expect the dispersed particles to re-approach each other precisely following the cyclotron period, causing rebunching at $\tau_{c}=1/f_{c}$.

The cartoons in Fig. 4(d) depict the processes starting from the time (*t′* = *t*−${\;\tau }_{p}$ = 0) when the particles are initially bunched to establish the strong signal $I_{tr,c}$. Here, we portray the particles with the blue dots. To capture the different initial velocities of the bunched emissions, four sets of cyclotron trajectories are plotted at two different radii. Such velocity dispersion leads to a quick decay in the signal as the particles move further away from each other from *t′* = 0 to *t′* = $\tau_{c}$/2. However, the particles re-approach each other after *t′* = $\tau_{c}$/2, resulting in rebunching at a period $\tau_{c}=1/f_{c}$. We confirm the experimental observations here to be entirely mediated by the cyclotron motions. This is supported by the results from an exhaustive survey of cyclotron echo dynamics, which indicates conclusively that ${1/\tau }_{c}$ depends linearly on *B* regardless of *F*, as illustrated in Fig. 4(e). We note that recently Zandi *et al.*^11^ reported a very similar periodic lensing effect, using a much smaller beam size (22 *μ*m in FWHM), where the cyclotron motion will go far beyond the initial plasma size, and is expected to be a pure electron cyclotron oscillation. Indeed, they observed a long-lived (>2 ns) oscillation without frequency evolution.

The echoing $I_{tr,c}$ observed here is reminiscent of the plasma and cyclotron echoes in studying the afterglow plasmas.^37–39^ Given the echoes can only survive when the microscopic phases of the periodic motions can be reconstructed, it is a powerful way to determine the microscopic dephasing time of the plasma systems.^40^ From the decay of $I_{tr,c}$ presented in Fig. 4(c), we obtain a dephasing time ≈700 ps, which is very similar to the decay time of the initial cyclotron wave observed.

## IV. SUMMARY AND OUTLOOK

In summary, we have demonstrated the multi-faceted capabilities of the new plasma imaging method using a UEM, which allows us to discover a novel cyclotron echo phenomenon and the staged non-linear wave mixing to set up the hybrid modes in the non-equilibrium system. Macroscopically, we find that the photoemitted electrons from micro-structured surfaces rapidly expand into a plasma disk and develop a transient cyclotron wave state under a strong magnetic field. Microscopically, the microplasma ejections form persistent cyclotron currents, which sustain the transient wave state over the dephasing time. The non-equilibrium cyclotron wave state here is a precursor state of the upper-hybrid resonance mode, which appears when the coherent mode is thermalized with the background gases and loses the granular features as observed in the decay of the echo signals. Future work is needed to elaborate on the particle–wave interactions relevant to the cyclotron dephasing, the wave generations, and the thermalization dynamics. These may be accomplished through informed theoretical analyses building on the conditions outlined by the current results and improved experiments targeting these problems.

From the technical perspective, the use of lower energy electrons (here 25 keV, which is nearly an order of magnitude smaller than the beam energy in a commercial TEM) is advantageous in gaining the field sensitivity. Ultimately, the image resolution is limited by the probe beam transverse emittance, which is estimated to be ∼0.02 mm⋅mrad.^28^ This gives a resolution at least on the 10 nm scale, as demonstrated by charactering the step-edge of the mesh; see the supplementary material. Time-compression of the pulses to 100 fs, however, leads to increased energy spread degrading the resolution to ∼100 nm. Reducing particle numbers in the electron pulses will decrease both the transverse and longitudinal emittances, leading to better performance at the cost of beam flux. We, thus, envision the highly flexible beam parameters and robust field-imaging protocol as demonstrated here to be very useful in efforts of directly resolving the fs/nm scale phenomena essential for studying microplasmas (or plasmons) and field-active devices impacting many areas,^5–7,9,13^ where dynamics initiated by the ultrashort laser pulses can be extremely short-lived but driven with higher local field/energy density.

## SUPPLEMENTARY MATERIAL

See the supplementary material for the electron optics settings, expansion of Maxwellian electron clouds, effective temperatures of photo-emitted electrons at high laser intensities, imaging plasma seeding dynamics, imaging collective plasma dynamics, and imaging plasma oscillations.

## Data Availability

The data that support the findings of this study are available from the corresponding authors upon reasonable request.

## References

[1] A. H. Zewail , “ Four-dimensional electron microscopy,” Science 328, 187–193 (2010). 10.1126/science.1166135 20378810

[2] W. E. King *et al.*, “ Ultrafast electron microscopy in materials science, biology, and chemistry,” J. Appl. Phys. 97, 111101 (2005). 10.1063/1.1927699

[3] O.-H. Kwon , V. Ortalan , and A. H. Zewail , “ Macromolecular structural dynamics visualized by pulsed dose control in 4D electron microscopy,” Proc. Natl. Acad. Sci. 108, 6026–6031 (2011). 10.1073/pnas.1103109108 21444766 PMC3076862

[4] M. T. Hassan , J. S. Baskin , B. Liao , and A. H. Zewail , “ High-temporal-resolution electron microscopy for imaging ultrafast electron dynamics,” Nat. Photonics 11, 425–430 (2017). 10.1038/nphoton.2017.79

[5] J. G. Eden *et al.*, “ Plasma science and technology in the limit of the small: Microcavity plasmas and emerging applications,” IEEE Trans. Plasma Sci. 41, 661–675 (2013). 10.1109/TPS.2013.2253132

[6] E. Forati , T. J. Dill , A. R. Tao , and D. Sievenpiper , “ Photoemission-based microelectronic devices,” Nat. Commun. 7, 13399 (2016). 10.1038/ncomms13399 27811946 PMC5097168

[7] J.-W. Han , D.-I. Moon , and M. Meyyappan , “ Nanoscale vacuum channel transistor,” Nano Lett. 17, 2146–2151 (2017). 10.1021/acs.nanolett.6b04363 28334531

[8] X. Fu *et al.*, “ Direct visualization of electromagnetic wave dynamics by laser-free ultrafast electron microscopy,” Sci. Adv. 6, eabc3456 (2020). 10.1126/sciadv.abc3456 33008895 PMC7852396

[9] M. L. Brongersma , N. J. Halas , and P. Nordlander , “ Plasmon-induced hot carrier science and technology,” Nat. Nanotechnol. 10, 25–34 (2015). 10.1038/nnano.2014.311 25559968

[10] P. Zhang and Y. Y. Lau , “ Ultrafast strong-field photoelectron emission from biased metal surfaces: Exact solution to time-dependent Schrödinger equation,” Sci. Rep. 6, 19894 (2016). 10.1038/srep19894 26818710 PMC4730214

[11] O. Zandi *et al.*, “ Transient lensing from a photoemitted electron gas imaged by ultrafast electron microscopy,” Nat. Commun. 11, 3001 (2020). 10.1038/s41467-020-16746-z 32532996 PMC7293293

[12] L. Chen *et al.*, “ Mapping transient electric fields with picosecond electron bunches,” Proc. Natl. Acad. Sci. 112, 14479–14483 (2015). 10.1073/pnas.1518353112 26554022 PMC4664376

[13] M. Centurion , P. Reckenthaeler , S. A. Trushin , F. Krausz , and E. E. Fill , “ Picosecond electron deflectometry of optical-field ionized plasmas,” Nat. Photonics 2, 315–318 (2008). 10.1038/nphoton.2008.77

[14] J. Li *et al.*, “ Ultrafast electron beam imaging of femtosecond laser-induced plasma dynamics,” J. Appl. Phys. 107, 083305 (2010). 10.1063/1.3380846

[15] C. M. Scoby , R. K. Li , and P. Musumeci , “ Effect of an ultrafast laser induced plasma on a relativistic electron beam to determine temporal overlap in pump-probe experiments,” Ultramicroscopy 127, 14–18 (2013). 10.1016/j.ultramic.2012.07.015 22951263

[16] R. K. Raman , Z. Tao , T. R. Han , and C. Y. Ruan , “ Ultrafast imaging of photoelectron packets generated from graphite surface,” Appl. Phys. Lett. 95, 181108 (2009). 10.1063/1.3259779

[17] M. Pant and L. K. Ang , “ Time-dependent quantum tunneling and nonequilibrium heating model for the generalized Einstein photoelectric effect,” Phys. Rev. B 88, 195434 (2013). 10.1103/PhysRevB.88.195434

[18] M. Buchanan , “ Everything is plasma,” Nat. Phys. 12, 394–394 (2016). 10.1038/nphys3756

[19] D. B. Melrose , *Instabilities in Space and Laboratory Plasmas* ( Cambridge University Press, 1986).

[20] W. P. Leemans *et al.*, “ GeV electron beams from a centimetre-scale accelerator,” Nat. Phys. 2, 696–699 (2006). 10.1038/nphys418

[21] B. S. Zerbe , X. Xiang , C. Y. Ruan , S. M. Lund , and P. M. Duxbury , “ Dynamical bunching and density peaks in expanding Coulomb clouds,” Phys. Rev. Accel. Beams 21, 064201 (2018). 10.1103/PhysRevAccelBeams.21.064201

[22] S. K. Hansen , S. K. Nielsen , M. Salewski , M. Stejner , and J. Stober , “ Parametric decay instability near the upper hybrid resonance in magnetically confined fusion plasmas,” Plasma Phys. Controlled Fusion 59, 105006 (2017). 10.1088/1361-6587/aa7978

[23] E. Kaselouris *et al.*, “ The influence of the solid to plasma phase transition on the generation of plasma instabilities,” Nat. Commun. 8, 1713 (2017). 10.1038/s41467-017-02000-6 29170379 PMC5700939

[24] C. Krafft and A. S. Volokitin , “ Electromagnetic radiation from upper-hybrid wave turbulence in inhomogeneous solar plasmas,” Plasma Phys. Controlled Fusion 62, 024007 (2020). 10.1088/1361-6587/ab569d

[25] P. H. Yoon , S. Kim , J. Hwang , and D.-K. Shin , “ Upper hybrid waves and energetic electrons in the radiation belt,” J. Geophys. Res.: Space Phys. 122, 5365–5376, 10.1002/2016JA023321 (2017).

[26] W. S. Kurth *et al.*, “ Electron densities inferred from plasma wave spectra obtained by the waves instrument on Van Allen probes,” J. Geophys. Res.: Space Phys. 120, 904–914, 10.1002/2014JA020857 (2015). 26167442 PMC4497465

[27] D. D. Ryutov and B. A. Remington , “ Scaling astrophysical phenomena to high-energy-density laboratory experiments,” Plasma Phys. Controlled Fusion 44, B407–B423 (2002). 10.1088/0741-3335/44/12B/328

[28] J. Williams *et al.*, “ Active control of bright electron beams with RF optics for femtosecond microscopy,” Struct. Dyn. 4, 044035 (2017). 10.1063/1.4999456 28868325 PMC5565489

[29] T. van Oudheusden *et al.*, “ Electron source concept for single-shot sub-100 fs electron diffraction in the 100 keV range,” J. Appl. Phys. 102, 093501 (2007). 10.1063/1.2801027

[30] F. Zhou , J. Williams , and C.-Y. Ruan , “ Femtosecond electron spectroscopy in an electron microscope with high brightness beams,” Chem. Phys. Lett. 683, 488–494 (2017). 10.1016/j.cplett.2017.03.019

[31] M. De Graef , in *Experimental Methods in the Physical Sciences*, edited by De Graef M. and Zhu Y. ( Academic Press, 2001), Vol. 36, pp. 27–67.

[32] M. Aeschlimann *et al.*, “ Observation of surface enhanced multiphoton photoemission from metal surfaces in the short pulse limit,” J. Chem. Phys. 102, 8606–8613 (1995). 10.1063/1.468962

[33] C. Cirelli *et al.*, “ Direct observation of space charge dynamics by picosecond low-energy electron scattering,” Europhys. Lett. 85, 17010 (2009). 10.1209/0295-5075/85/17010

[34] J. G. Fujimoto , J. M. Liu , E. P. Ippen , and N. Bloembergen , “ Femtosecond laser interaction with metallic tungsten and nonequilibrium electron and lattice temperatures,” Phys. Rev. Lett. 53, 1837–1840 (1984). 10.1103/PhysRevLett.53.1837

[35] Y. Zhou and P. Zhang , “ A quantum model for photoemission from metal surfaces and its comparison with the three-step model and Fowler–DuBridge model,” J. Appl. Phys. 127, 164903 (2020). 10.1063/5.0004140

[36] J. Portman *et al.*, “ Computational and experimental characterization of high-brightness beams for femtosecond electron imaging and spectroscopy,” Appl. Phys. Lett. 103, 253115 (2013). 10.1063/1.4855435

[37] Y. W. Hou , Z. W. Ma , and M. Y. Yu , “ The plasma wave echo revisited,” Phys. Plasmas 18, 012108 (2011). 10.1063/1.3533447

[38] L. O. Bauer , F. A. Blum , and R. W. Gould , “ Plasma echoes at upper hybrid resonance,” Phys. Rev. Lett. 20, 435–439 (1968). 10.1103/PhysRevLett.20.435

[39] R. M. Hill and D. E. Kaplan , “ Cyclotron resonance echo,” Phys. Rev. Lett. 14, 1062–1063 (1965). 10.1103/PhysRevLett.14.1062

[40] R. W. Gould , T. M. O'Neil , and J. H. Malmberg , “ Plasma wave echo,” Phys. Rev. Lett. 19, 219–222 (1967). 10.1103/PhysRevLett.19.219

